# The value of screening in patient populations with high prevalence of a disorder

**DOI:** 10.1186/1741-7015-12-14

**Published:** 2014-01-28

**Authors:** David Goldberg

**Affiliations:** 1 Institute of Psychiatry, King’s College, London, UK

**Keywords:** Screening, Depression, Positive predictive value, Low and middle income countries

## Abstract

Thombs and colleagues have shown that screening consecutive attendees in primary care settings in high income countries for depression is not worthwhile. However, it is dangerous to generalize from high income countries such as the USA to the rest of the world. The positive predictive value of any screening test for depression is affected by the prevalence of the disorder in the population being considered. Populations with an increased prevalence of depression, such as those with chronic physical disorders, or with a history of depression or other mental health problems may benefit from screening, even in high income countries. Populations in low and middle income countries (LMIC) may also benefit from screening if they are experiencing severe social adversity, including poverty. Two examples are given, in which screening with a brief screening questionnaire was followed by collaborative stepped care, to the considerable benefit of the patients in LMIC.

Please see related article: http://www.biomedcentral.com/1741-7015/12/13.

## Main text

Thombs and colleagues [1] have brought the argument about screening consecutive attendees for depression up to date, but there is really nothing new in their general conclusion that in high income countries there are no positive gains in the use of screening tests for depression. They are correct in saying that the advice of the US Public Health Service, stating that family physicians should use such screening tests, is not supported by the available evidence. It has been known since 2001 that many studies that merely provided general practitioners with feedback of high scores on screening questionnaires, have not resulted in increased recognition [2], and even those studies that did report increased recognition did not lead to better treatment [3,4]. Indeed, similar findings have been reported from much earlier studies in primary care [5]. The first National Guideline of Depression in the UK in 1993 [6] did not recommend routine screening, but did recommend that screening should be undertaken in primary care and general hospital settings for depression in high risk groups; for example, those with a history of depression, significant physical illnesses causing disability, or other mental health problems, such as dementia. The guideline was re-issued in 1999 [7] with substantially the same advice, advising clinicians to be alert to possible depression (particularly in people with a history of depression or a chronic physical health problem with associated functional impairment) and consider asking people who may have depression the two screening questions that are used to define depression in both the International Classification of Disease [8] and the *Diagnostic and Statistical Manual* (DSM) system of the American Psychiatric Association [9].

## The positive predictive value of a screening test

The original National Institute for Health and Clinical Excellence (NICE) guideline for depression [6] concluded that high prevalence populations could be screened with much better results. This is because the proportion of high scorers on a test which turn out to be true cases (the positive predictive value, or PPV) is greatly affected by the point prevalence of the disorder in the screened population [10]. This is true of any screening test, including the nine-item Patient Health Questionnaire (PHQ-9) recommended by the UK Department of Health.

Thus, at 10% prevalence, a test with a sensitivity of 90% and a specificity of 85% would have a PPV of only 40%, so a clinician could be forgiven for not wishing to be influenced by a test with such a low chance of being correct. However, the same test at a prevalence of 45% would have a PPV of 83.5%. The negative predictive value is affected to a much smaller degree, being 98.7% at 10% prevalence, but is a still acceptable 91.2% at a prevalence of 45%. It can be seen that in high prevalence populations, the same screening test works very much better than it does in predominantly healthy populations. For example, Whooley *et al*. [11] argue that screening is worthwhile in all patients with chronic cardiac disease.

## Low and middle income countries

Many populations of people seeking care in low and middle income countries (LMIC) have a high prevalence of psychological disorders: for example, in the World Health Organization study [12] of Mental Illness in General Health Care, the overall point prevalence of disorders was 52.5% in Santiago de Chile, and 35.5% in Rio de Janeiro, but below 10% in Verona, Ibadan, Nagasaki, and Shanghai, and only 11.9% in Seattle. In both of the South American cities, there was considerable poverty and social adversity to add to the list of features known to be associated with high prevalence.

Of course, these high prevalences do not apply to all settings in LMIC, but they are common wherever social adversity and poverty are marked, so that use of screening tests is widespread in these countries. In recent years, some of the older tests such as the 12-item General Health Questionnaire (GHQ-12) [13-15], the Symptom Rating Questionnaire [16], and the Hospital Depression and Anxiety Scales (HADS) [13] have been used, as well as more recent instruments such as the PHQ-9 [17] and the two Kessler scales (K6 and K10) [18,19]. In general, there is agreement that in these settings, the shorter scales are as effective as the longer ones [20].

However, two further things are also necessary, other than a high prevalence of depression in the population to be screened, and these involve both healthcare provider and patient The doctor must have the ability to both diagnose and assess the severity of the depression, and must be able to provide effective treatment. It is also helpful if the doctor is perceived by the patient as someone with whom it is easy to communicate. The patient must be prepared to admit that they have a need for an intervention, be allowed to choose what treatment they receive, and to invest both time and cooperation in such treatment.

## Two examples of successful use of screening tests in LMIC

Both of these examples [14,15] used screening with the GHQ-12 followed by the use of collaborative stepped care (CSC), and compared care by a lay mental health worker with the condition of usual care by the primary care clinician, in a randomized controlled trial. In the study by Patel *et al*., the CSC condition meant that all patients with high scores on a screening questionnaire were given psycho-education by the lay counsellors, but in patients with higher scores this was supplemented by an antidepressant by the primary care physician. Psycho-education taught patients strategies to alleviate their symptoms, using techniques such as breathing exercises for anxiety symptoms and scheduling activities for symptoms of depression. Moderately or severely ill patients who were offered drug treatment could also have group interpersonal psychotherapy from the lay health worker. A later paper [21] followed these patients up for 12 months, and found a 30% decrease in the prevalence of common mental disorders among those with baseline ICD-10 diagnoses (risk ratio (RR) = 0.70, 95% CI 0.53 to 0.92); and a similar effect among the subgroup of participants with depression (RR = 0.76, 95% CI 0.59–0.98). There was a 36% reduction in suicide attempts/plans over 12 months (RR = 0.64, 95% CI 0.42 to 0.98) among baseline ICD-10 cases. Strong effects were observed on days out of work and psychological morbidity, and modest effects on overall disability.

In the study by Araya *et al*. [15], low-income female patients with severe depression in Santiago, Chile, were allocated to usual care or to care administered by a 3-month, multi-component intervention led by a non-medical health worker. At six months’ follow-up, 70% of the intervention group compared with 30% of the usual care group, had recovered. A later paper [22] considered outcome at 6 months and found that, after adjusting for initial severity, women receiving the stepped-care program had a mean of 50 additional depression-free days over 6 months relative to patients allocated to usual care. The CSC program was marginally more expensive than usual care (an extra 216 Chilean pesos per depression-free day). The authors concluded that small investments to improve depression appear to yield larger gains in poorer environments.

## Future directions and conclusions

It is to be hoped that future studies in all populations in which an increased prevalence of depression may be expected will continue to use brief screening procedures to detect depression. In LMIC, CSC offered by a trained non-medical health worker has been shown to produce excellent results.

## Competing interests

The author is responsible for the General Health Questionnaire, and shares the Copyright with the Institute of Psychiatry, King’s College, London, UK.

## Authors’ information

The author is Chairman of the Primary Care Consultation Group for ICD-11 of the World Health Organization.
